# IKKα Induces Epithelial–Mesenchymal Changes in Mouse Skin Carcinoma Cells That Can Be Partially Reversed by Apigenin

**DOI:** 10.3390/ijms23031375

**Published:** 2022-01-25

**Authors:** Verónica A. García-García, Josefa P. Alameda, Angustias Page, Antonio Mérida-García, Manuel Navarro, Adrián Tejero, Jesús M. Paramio, Rosa A. García-Fernández, M. Llanos Casanova

**Affiliations:** 1 Molecular and Translational Oncology Unit, Centro de Investigaciones Energéticas, Medioambientales y Tecnológicas (CIEMAT), 28040 Madrid, Spain; VeronicaArantxa@externos.ciemat.es (V.A.G.-G.); pilar.alameda@ciemat.es (J.P.A.); a.page@ciemat.es (A.P.); ajmerida@saludcastillayleon.es (A.M.-G.); manuel.navarro@ciemat.es (M.N.); Adrian.Tejero@ciemat.es (A.T.); jesusm.paramio@ciemat.es (J.M.P.); 2 Biomedical Research Institute I+12, 12 de Octubre University Hospital, 28040 Madrid, Spain; 3 Centro de Investigación Biomédica en Red de Cáncer (CIBERONC), 28029 Madrid, Spain; 4 Complejo Asistencial de Zamora, 49022 Zamora, Spain; 5 Department of Animal Medicine and Surgery, Facultad de Veterinaria, Complutense University of Madrid (UCM), 28040 Madrid, Spain; ragarcia@ucm.es

**Keywords:** IKKα, skin carcinoma cells, EMT changes, cell survival, cell migration, apigenin

## Abstract

NMSC (non-melanoma skin cancer) is a common tumor in the Caucasian population, accounting for 90% of skin cancers. Among them, squamous cell carcinomas (SCCs) can metastasize and, due to its high incidence, constitute a severe health problem. It has been suggested that cutaneous SCCs with more risk to metastasize express high levels of nuclear IKKα. However, the molecular mechanisms that lead to this enhanced aggressiveness are largely unknown. To understand in depth the influence of nuclear IKKα in skin SCC progression, we have generated murine PDVC57 skin carcinoma cells expressing exogenous IKKα either in the nucleus or in the cytoplasm to further distinguish the tumor properties of IKKα in both localizations. Our results show that IKKα promotes changes in both subcellular compartments, resembling EMT (epithelial–mesenchymal transition), which are more pronounced when IKKα is in the nucleus of these tumor cells. These EMT-related changes include a shift toward a migratory phenotype and induction of the expression of proteins involved in cell matrix degradation, cell survival and resistance to apoptosis. Additionally, we have found that apigenin, a flavonoid with anti-cancer properties, inhibits the expression of IKKα and attenuates most of the pro-tumoral EMT changes induced by IKKα in mouse tumor keratinocytes. Nevertheless, we have found that apigenin only inhibits the expression of the IKKα protein when it is localized in the cytoplasm.

## 1. Introduction

NMSC is the most common human neoplasm in the Caucasian population, accounting for 90% of skin cancers; moreover, the incidence of both benign and malignant NMSC has been increasing at an alarming rate in recent years, including in the population younger than 40 years [1], making NMSC an important health problem.

NMSC includes two different entities: BCCs (basal cell carcinomas) and SCCs; while BCCs rarely metastasize, metastasis from high-risk SCC are more frequent and may be lethal. Almost 5% of SCCs metastasize; therefore, due to its high incidence, the mortality concomitant to aggressive cutaneous SCCs is reaching important numbers [2]. Furthermore, the risk for developing skin SCCs highly increases in several conditions, i.e., almost all patients suffering from recessive dystrophic epidermolysis bullosa (RDEB) are prone to develop aggressive and metastatic skin SCCs at early ages [3]; in addition, organ transplant recipients have a higher risk to develop skin SCCs (65–250 times compared with the general population) that in addition are more aggressive [4]. This makes the elucidation of the molecular changes that lead to aggressive skin SCCs an urgent matter. In this regard, it has been reported that the nuclear localization of IKKα (inhibitor of nuclear factor kappa-B kinase subunit alpha) is a hallmark of aggressive human cutaneous SCCs with a high risk to metastasize [5]. However, despite these data, the mechanisms through which IKKα favors cutaneous SCCs aggressiveness have not been fully unveiled.

In the cytoplasm, IKKα is part of the IKK complex that regulates the NF-κB transcription factor [6]. It is considered that its main function is the regulation of epidermal differentiation, since *IKKα*-deficient mice exhibit marked morphological abnormalities that include taut and shiny skin that is unable to differentiate, and they lack a barrier function; as a consequence, *IKKα*-null mice die shortly after birth by dehydration [7,8]. In addition to its cytoplasmic functions, nuclear roles for IKKα have been identified [9,10,11]; thus, it was reported that it regulated the epidermal differentiation in the nucleus, independent of the NF-κB pathway and through the modulation of the EGFR and Smads pathways [12,13,14]. Therefore, due to its key role in skin morphogenesis, it is not surprising that IKKα was also involved in NMSC development and progression [5,15,16,17,18]. Furthermore, our group has recently described that IKKα may act in both subcellular localizations, in the nucleus and in the cytoplasm of keratinocytes, as a promoter of skin SCC aggressiveness [15].

A well-recognized mechanism that promotes cancer progression and malignancy is the EMT process, since it enhances the invasive properties of cancer cells, including epidermal cells [19,20]. EMT causes the reprogramming and phenotypic changes through which immobile cancer epithelial cells acquire a migratory mesenchymal phenotype and repress the expression of epithelial markers such as keratins, while inducing those characteristics of mesenchymal cells, such as Vimentin [21,22]. According to these changes, EMT is accompanied by the induction of the expression of metalloproteases, such as MMP9, that can degrade the basement membrane, thereby favoring cell migration and invasion [23]. Notably, the EMT process not only induces increased cancer cell motility and invasiveness but also allows cancer cells to avoid anoikis and cellular senescence while favoring cell survival [24,25]. Furthermore, it has been suggested that EMT facilitates the acquisition of resistance to apoptosis, a key characteristic required for tumor survival [25], which is mainly mediated by Snail1, a master regulator of EMT [26,27]. Moreover, EMT plays a role not only in tumor progression, but also in the metastatic cascade of many solid tumors, representing a hallmark of this event [28]. EMT is also implicated in tumor resistance to chemotherapy and radiotherapy [28,29].

In the case of NMSC, there is evidence of EMT involvement in the progression of actinic keratosis to invasive cutaneous SCC [30]. EMT activation has also been found in invasive skin SCCs compared to normal skin and with SCCs in situ [31]. Therefore, given that EMT and nuclear expression of IKKα are two factors that favor the greater aggressiveness of skin SSCs [5], in this work, we have studied whether nuclear IKKα induces EMT in epidermal cancer cells. For this purpose, we have generated PDVC57 skin carcinoma cells that constitutively express increased levels of IKKα in the nucleus or in the cytoplasm (named as C57-N-IKKα and C57-C-IKKα cells respectively). Our results show that both types of PDVC57 cells that express ectopic IKKα acquire a high capacity of migration and induce the expression of MMP9 and the survival proteins Bcl2 and Akt, while repressing the expression of proapoptotic proteins. These changes suggest that IKKα favors the EMT process in tumor epidermal cells. This effect is particularly marked in the C57-N-IKKα cells, which in addition, switch their epithelial morphology toward a fibroblastoid phenotype, induce the expression of *Snail1*, and repress that of keratin 5 (K5).

Additionally, and given that the finding of an efficient treatment for aggressive skin SCCs is a critical issue, we have tested the possible utility of apigenin as a potential anti-skin SCC treatment. Apigenin is a bioactive flavonoid with anticancer activity in many types of cancers whose action is mediated by different mechanisms [32]. In the case of prostate cancer, it was shown that apigenin exerted its antitumor effect through direct interaction with IKKα [33,34]. Although it has been shown that apigenin also acts as an antitumor agent in NMSC [35,36], its mechanisms of action remain largely unknown, and the possible involvement of IKKα in the anticancer activity of apigenin in skin SCCs has not been addressed yet. Thus, we have carried out this study and found that apigenin inhibits the expression of the IKKα protein, as well as the EMT changes induced by IKKα in the C57 cells, at low concentrations and at early time points of treatment, but only when IKKα is localized in the cytoplasm of keratinocytes.

## 2. Results

### 2.1. Exogenous Expression of IKKα in the Nucleus or Cytoplasm of PDVC57 Skin Carcinoma Cells

**Figure 1:**
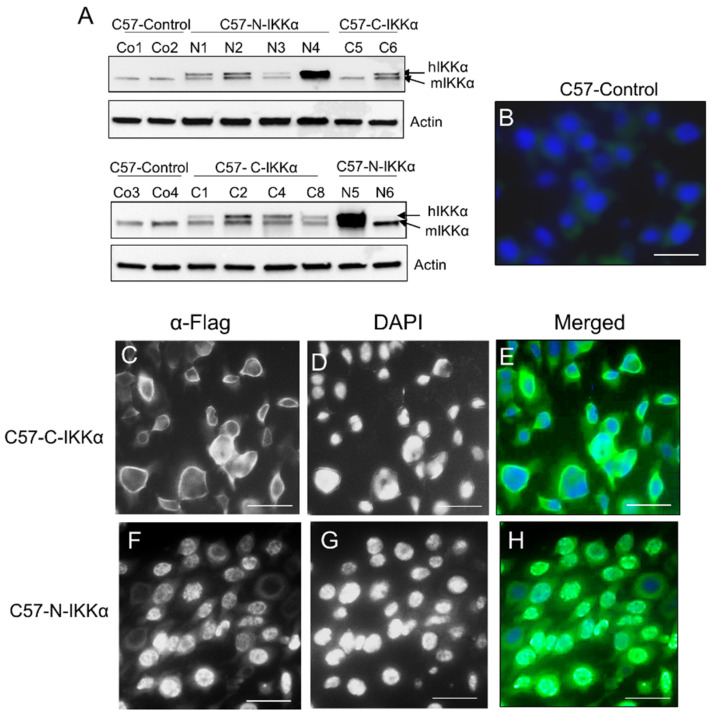
Generation of the C57-C-and N-IKKα clones. (**A**) Western blots showing the expression of the exogenous IKKα in the C57-C–and-N-IKKα cells (upper arrow). The expression in different selected clones is shown. Actin was used as loading control. hIKKα: exogenous human IKKα protein; mIKKα: endogenous mouse IKKα protein. (**B**–**H**) Immunofluorescence analysis of the expression of the transgenic protein in the C57-Control, C57-C-IKKα and C57-N-IKKα cells. An anti-Flag antibody was used. (**B**) In the C57-Control cells, transfected with the empty vector, Flag is not detected. (**C**,**E**) Cytoplasmic expression of the Flag reporter in the C57-C-IKKα cells transfected with the β-actin-C-IKKα construct. (**F**,**H**) Nuclear expression of Flag is observed in C57-N-IKKα cells transfected with the β-actin-N-IKKα construct. (**D**,**G**) Dapi stainings mark the nuclei. Scale bar: 40 μm.

We have generated transfectant cells which express an exogenous human IKKα protein specifically in the cytoplasmic or nuclear compartment of the PDVC57 cell line (designated as C57-C-IKKα and C57-N-IKKα cells, respectively). PDVC57 mouse skin carcinoma cells were chosen because they bear the *Ha-ras* mutation carried by most of the chemically induced mouse skin tumors and some human skin carcinomas [37,38,39,40]. PDVC57 cells were stably transfected with the human IKKα complementary DNA under the control of the β-actin promoter, which lacks the nuclear localization signal (NLS) in the C-IKKα construct or which carries an extra NLS in the N-IKKα construct, and both of them tagged with a Flag epitope (Figure 1A) or with the empty vector [15]. Pooled of stable transfectants from approximately 50 different G418-resistant colonies were used to minimize any potential effect of clonal selection. Results from C57-C-IKKα and C57-N-IKKα cells were compared with that of C57-Control cells (which carry the empty vector). Western blot showed that C57-C-IKKα and C57-N-IKKα cells express the transgene, which is slightly larger due to the tag (Figure 1A). Next, we performed immunofluorescence staining to check in situ the expression of the transgene (Figure 1B–H). We found that C57-C-IKKα (Figure 1C–E) and C57-N-IKKα cells (Figure 1F–H) expressed the transgene in the cytoplasm and in the nucleus, respectively. For the following experiments, we chose two clones of each genotype which expressed different levels (low or high) of the transgene; therefore, clones N3 and N4 carrying the N-IKKα construct and clones C4 and C8, bearing the C-IKKα construct were selected; in the case of Control cells, clones Control1 and Control2 were chosen for studies.

### 2.2. Increased Migratory Capacity of Both C57-C-IKKα and C57-N-IKKα Cells in Culture

**Figure 2:**
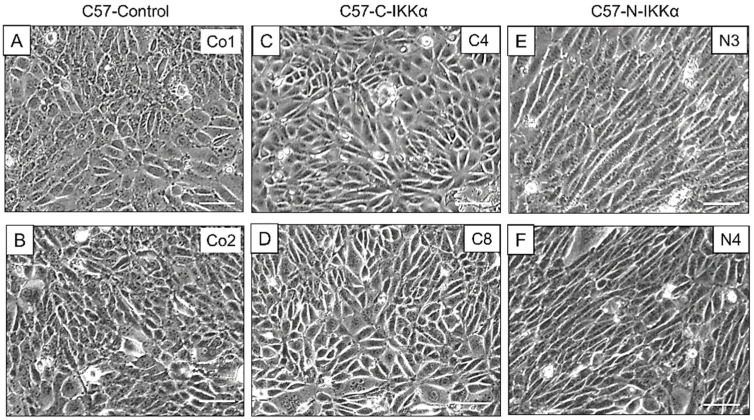
Appearance of C57 cells of each genotype growing in culture. (**A**–**F**) Representative images of two different clones of C57 cells of the three genotypes growing for 72 h. Note the contrast between the epithelial morphology of C57-Control (**A**,**B**) and C57-C-IKKα (**C**,**D**) cells and the fibroblastoid morphology of C57-N-IKKα cells (**E**,**F**). Co1, Co2: C57-Control 1 and C57-Control 2 clones, respectively; C4, C8: C57-C-IKKα clones C4 and C8, respectively; N3, N4: C57-N-IKKα clones N3 and N4, respectively. Scale bars: 50 μm.

**Figure 3:**
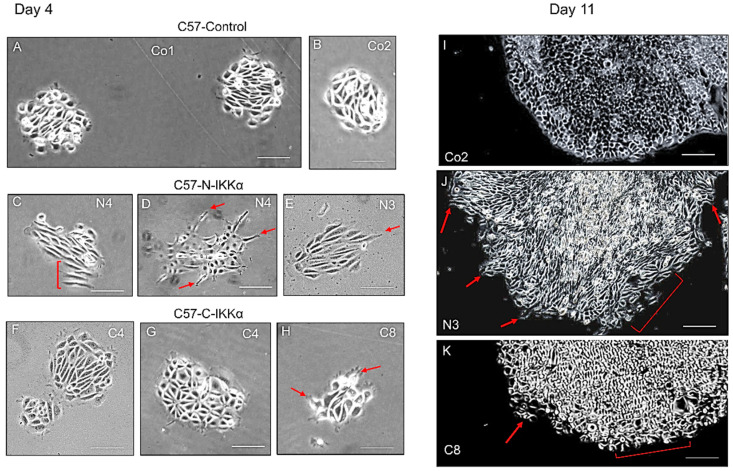
Morphological changes of the colonies formed by C57 cells expressing the ectopic IKKα. Representative images showing the appearance of the colonies formed by C57 cells of the three genotypes four (**A**–**H**) and eleven (**I**–**K**) days after seeding. Observe that C57-Control cells form rounded and compact colonies with a well-defined border (**A**,**B**). By contrast, colonies formed by C57-N-IKKα cells appear loose, with cells disposed in parallel (bracket) and show the presence of migratory cells moving away from the core of the colony (red arrows) (**D**,**E**). Colonies formed by C57-C-IKKα cells show an intermediate phenotype between that of C57-Control and C57-N-IKKα cells: occasional migratory cells in the periphery of the colonies are detected (red arrows) (**F**–**H**). (**I**–**K**) At eleven days post seeding, foci of migratory cells were detected (red arrows) in C57-N-IKKα (**J**) and C57-C-IKKα (**K**) cells. Bracket in (**J**) points to foci of loose cells. Co1, Co2: C57-Control 1 and C57-Control 2 clones, respectively; C4, C8: C57-C-IKKα clones C4 and C8, respectively; N3, N4: C57-N-IKKα clones N3 and N4, respectively. Scale bars: (**A**–**H**) 50 μm; (**I**–**K**) 150 μm.

We observed that the expression of IKKα in the nucleus of C57-N-IKKα cells confers them an enlarged and elongated shape that reminds that of fibroblasts (Figure 2). Therefore, we performed colony-forming assays to better analyze the shape of the colonies formed by the three types of cells and found that, in contrast with the rounded and dense colonies with a well-defined shape formed by C57-Control cells (Figure 3A,B), those formed by C57-N-IKKα cells appeared large, loose and showed an imprecise contour, with cells that did not compact and did not acquire the classic rounded form characteristic of cell colonies (Figure 3C–E); in contrast, colonies of C57-C-IKKα cells usually had a phenotype similar to that of C57-Control cells (Figure 3F), although colonies of an intermediate phenotype between that of C57-Control and C57-N-IKKα cells were sometimes perceived (Figure 3G,H). When observed at higher magnification, the presence of groups of cells migrating from the C57-N-IKKα colonies (Figure 3J) is observed; these groups of cells were also detected in the C57-C-IKKα colonies, although they were less frequent (Figure 3K).

**Figure 4:**
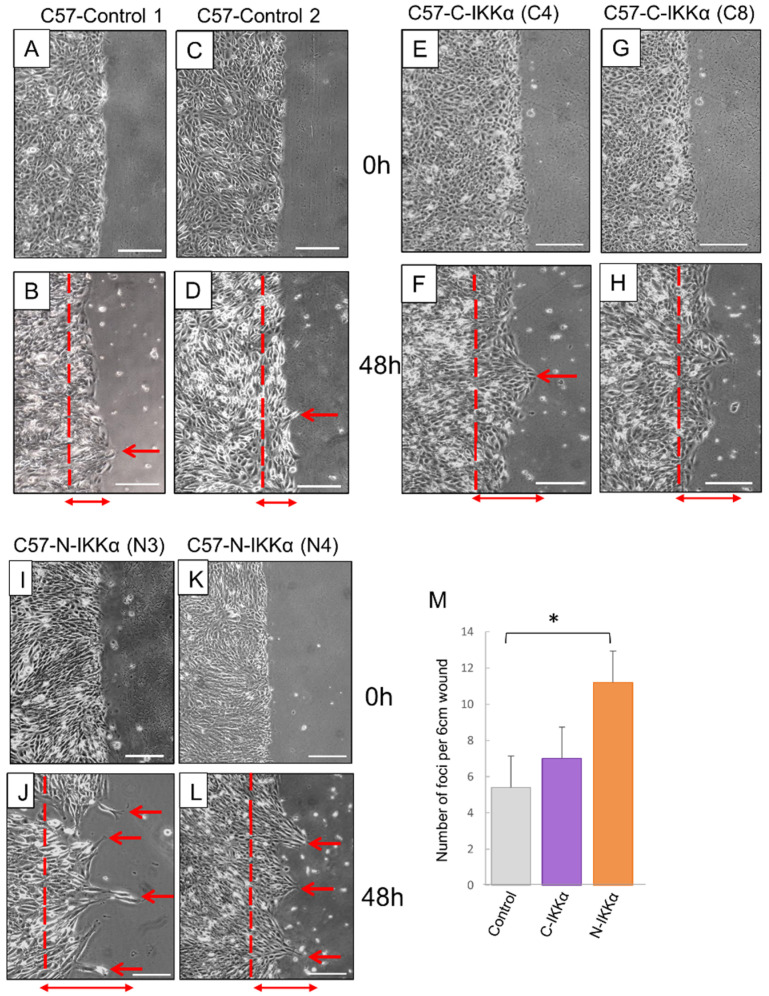
Wound healing assay. Cell pictures at the time of the scratch (0 h) of the two selected clones of each genotype are presented (**A**,**C**,**E**,**G**,**I**,**K**); below, migration 48 h after the scratch wound is shown (**B**,**D**,**F**,**H**,**J**,**L**). Note the increased number of foci of migrating cells of both the C57-N- and-C-IKKα cells (red arrows) (**F**,**H**,**J**,**L**); also the migration distance is longer in C57 cells expressing the exogenous IKKα (double head red arrows); discontinued red lines indicate the position in which the migration initiates in each cell type. In C57-Control cells, few migratory cells are detected (**B**,**D**). (**M**) Graphic representation of the number of foci of migration found per 6 cm wound length. Data are shown as mean ± SEM; * *p* < 0.05 by t test. Scale bars: (**A**,**C**,**E**,**G**,**I**,**K**) 100 μm; (**B**,**D**,**F**,**H**,**J**,**L**) 70 μm.

Next, the effect of nuclear and cytoplasmic IKKα expression on C57 cell motility was analyzed in in vitro wound healing assays: cells growing in monolayer cultures were subjected to scratch wounds and found that at 48 h post-scratching both C57-C-and-N-IKKα cells formed frequent peaks of migration, (Figure 4E–L); by contrast, in the scratch wound of the C57-Control cells, only a few migratory cells were observed (Figure 4A–D). Additionally, both C57-N-and-C-IKKα cells achieved greater migration distance (Figure 4E–L). Therefore, our results suggest that increased levels of IKKα lead to an enhancement of the capacity of migration of tumor epidermal cells, and importantly, similar results were obtained from the analysis of clones that express low or high levels of ectopic IKKα, suggesting that it is sufficient a small increment in the expression level of IKKα to induce the indicated changes (Figure 3 and Figure 4).

### 2.3. Induction of Snail and Vimentin Expression and Inhibition of K5 in C57-N-IKKα Cells

**Figure 5:**
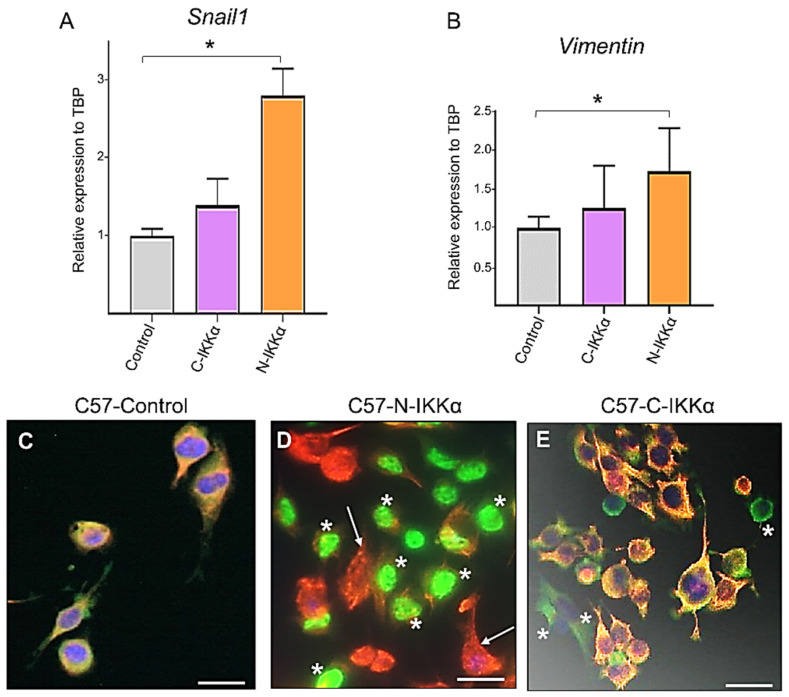
Induction in the C57-N-IKKα cells of the expression of the EMT master gene *Snail1* and repression of the K5 epithelial marker. (**A**) The analysis by qPCR of the expression of *Snail1* shows that it is induced in the C57-N-IKKα cells (arbitrary units). (**B**) qPCR analysis showing the increased expression of Vimentin in the C57-N-IKKα cells (arbitrary units). Data are shown as mean ± SEM; * *p* < 0.05 by two-tailed unpaired t test with Welch’s correction. (**C**–**E**) Immunofluorescence staining with anti-IKKα (green) and anti-K5 (red) antibodies. (**C**) C57-Control cells show co-expression of both IKKα and K5 in each cell. (**D**) Observe that in the C57-N- IKKα cells, most cells express IKKα in the nucleus (green) and do not express K5 (asterisks), and on the contrary, those scarce cells that do not express nuclear IKKα express K5 (arrows). (**E**) Both, IKKα and K5, are co-expressed in almost all C57-C-IKKα cells, although sporadically it can be observed the presence of cells that express IKKα in the cytoplasm but do not express K5 (asterisks). Scale bars: 35 μm.

The fibroblastoid phenotype of C57-N-IKKα cells together with their increased migratory ability suggest that they were undergoing a process of EMT; therefore, we analyzed the expression of the master gene of EMT, *Snail1*, and found that it was highly induced in C57-N-IKKα cells compared with C57-Control cells, while only a slight increase in the expression of *Snail1* (not statistically significant) was detected in the C57-C-IKKα cells (Figure 5A). Another feature of EMT is a switch of the expression of epithelial markers toward mesenchymal ones; therefore, we analyzed the expression of Vimentin and found that it was increased in C57-N-IKKα cells (Figure 5B), which correlated with the morphological change observed in the shape of these cells. Next, we examined the expression of the keratin K5 in the three types of C57-cells, since the downregulation of the expression of keratins in epithelial cells is a well-established biomarker of EMT [41]. We found that the expression of K5 was inhibited in the C57-N-IKKα cells: it was observed that those cells expressing the ectopic IKKα did not express K5 (Figure 5D); thus, we only detected K5 expression in the minority of cells, which do not express the ectopic nuclear IKKα (arrow in Figure 5D). By contrast, co-expression of both K5 and IKKα was detected in both C57-Control and C57-C-IKKα cells (Figure 5C,E).

**Figure 6:**
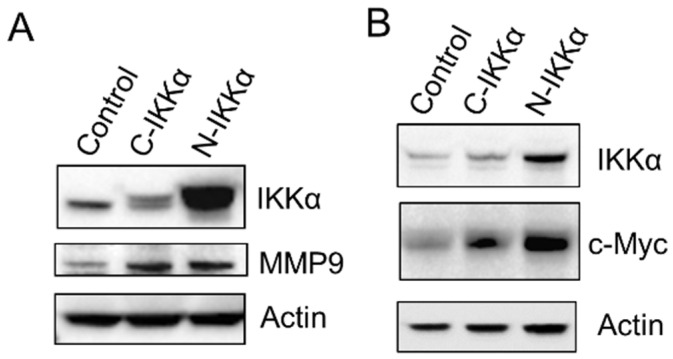
Induction of MMP9 and c-Myc expression in PDVC57 cells expressing ectopic IKKα. (**A**,**B**) WBs of total protein extracts from C57-Control and C57-C- and-N-IKKα cells showing that the levels of MMP9 (**A**) and c-Myc (**B**) are increased in both type of cells that express the exogenous IKKα protein.

Another change that occurs during the EMT process is the induction of the expression of metalloproteases that can degrade the basement membrane, thereby favoring cell migration and invasion. We analyzed MMP9 expression and saw that it was induced in both C57-C-IKKα and C57-N-IKKα cells (Figure 6A), which is consistent with the increased migratory capacity of both types of cells (Figure 4). To determine the source of MMP9 production, we analyzed the expression of c-Myc since it has been reported that Myc induces MMP9 [42], and c-Myc overexpression is involved in EMT during transformation of different epithelial cells [43]. The analysis by WB showed increased levels of c-Myc in the C57-N-IKKα cells and a slight induction in the C57-C-IKKα ones (Figure 6B). These results agree with our previous results, which showed the induction of c-Myc both in skin carcinomas developed by chemical skin carcinogenesis in K5-N-IKKα transgenic mice [15] and in the N-H460-IKKα lung adenocarcinoma cells [44].

We confirmed these results in another cell line, the mouse tumor keratinocyte PB cell line [45] (Supplementary Figure S1). PB cells were transfected with the C-or-N-IKKα constructs (or with the empty vector), and it was obtained three pooled stable transfectans clones from 25–40 different colonies, named as PB-C-IKKα, PB-N-IKKα and PB-Control clones. The correct expression of the transgene in the nucleus of PB-N-IKKα cells and in the cytoplasm of the PB-C-IKKα cells was determined by immunofluorescence analysis (Figure S1A–C). Western blot analysis confirmed the expression of the exogenous human IKKα in the transfected PB-C-and-N-IKKα cells (Figure S1D). Similar to the data obtained with the C57 cells, we observed that both PB-C-and-N-IKKα cells expressed increased levels of MMP9 and c-Myc; additionally, it was found that the expression of K5 was inhibited in the PB-N-IKKα cells, since similar to the staining in the C57-N-IKKα cells, those cells expressing the ectopic IKKα did not express K5 (asterisks in Figure S1E), while K5 expression was observed in the cells which do not express the ectopic nuclear IKKα (arrows in Figure S1E). Likewise, equal to the staining observed in C57-C-IKKα cells, co-expression of both K5 and IKKα was detected in PB-C-IKKα cells (Figure S1F). Therefore, these results suggest that the changes in both C57 and PB-C-and-N-IKKα tumor keratinocytes are likely due to augmented IKKα expression in each subcellular localization of skin carcinoma cells.

### 2.4. C57-N-IKKα Cells Have Increased Survival Capacity

**Figure 7:**
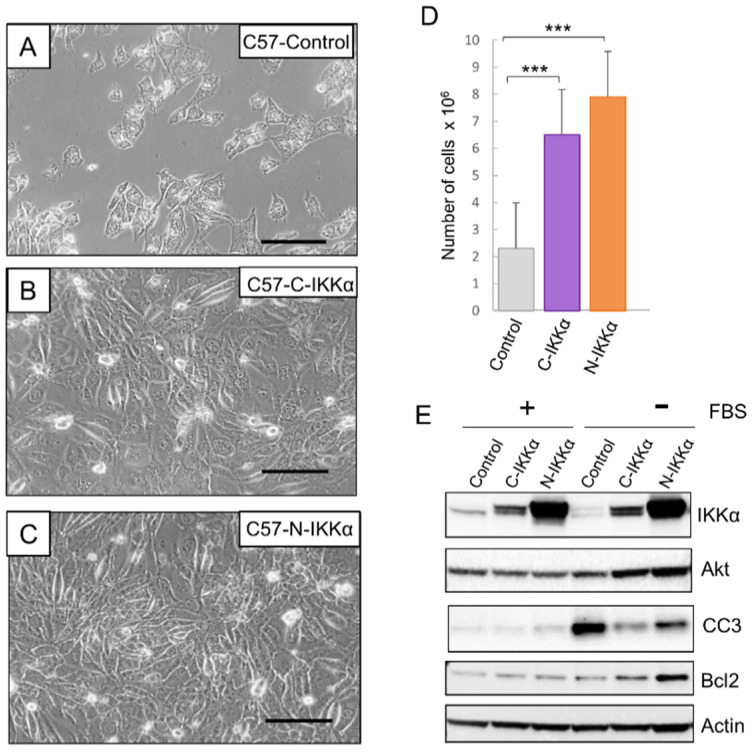
Increased survival capacity of C57-N-IKKα cells in a stressful situation. (**A**–**C**) Representative experiment of the appearance of cells after 7 days of growth in the absence of serum. Observe the diminished number of C57-Control cells (an average of 2.3 × 10^6^ ± 0.4 × 10^6^ cells/p100) versus 6.5 × 10^6^ ± 0.4 × 10^6^ and 7.9 × 10^6^ ± 1.2 × 10^6^ of C57-C-IKKα and C57-N-IKKα cells/p100, respectively (**D**) Graphical representation of the number of cells that survive after 7 days of growth in the absence of serum (*** *p* < 0.001; data are mean ± SEM; by t-test). Scale bars: 50 μm. (**E**) WB of total protein extracts from C57-Control and C57-C- and-N-IKKα cells showing the induction of the survival proteins Akt and Bcl2 in the C57 cells that express the ectopic IKKα and the lower than control amount of the pro-apoptotic cleaved-Caspase 3 (CC3) in both C57-C-IKKα and C57-N-IKKα cells.

Another characteristic of the cells that experiment an EMT process is the increase in their survival capacity as well as in their apoptotic resistance. Thus, we tested the behavior of the C57 cells of the three genotypes in a situation of stress such as culture in the absence of serum. It was observed that both C57-C-IKKα and C57-N-IKKα cells had an increased capacity to survive in absence of FBS, being the number of cells that persisted after 7 days of culture in the absence of FBS higher in the C57 cells expressing the exogenous IKKα in each subcellular localization (Figure 7A–D). In agreement with this observation, we analyzed the levels of expression of Akt, a protein known to increase the survival of cancer cells [46] and found that it was induced in both types of C57-IKKα cells (Figure 7E), while a lower than control amount of the proapoptotic cleaved-Caspase 3 was detected in both C57-C-IKKα and C57-N-IKKα cells (Figure 7E). Next, we analyzed the expression of the anti-apoptotic and pro-survival protein Bcl-2 and found that its expression was also increased in both types of C57-IKKα cells, mainly in the C57-N-IKKα cells (Figure 7E). Therefore, our results show that the increased levels of IKKα in the C57 skin carcinoma cells confers them a greater capacity for survival and resistance to apoptosis in stressful situations, such as culture in the absence of serum.

### 2.5. Apigenin Inhibits the Expression of the IKKα Protein in the Cytoplasm but Not in the Nucleus of C57 Cells

**Figure 8:**
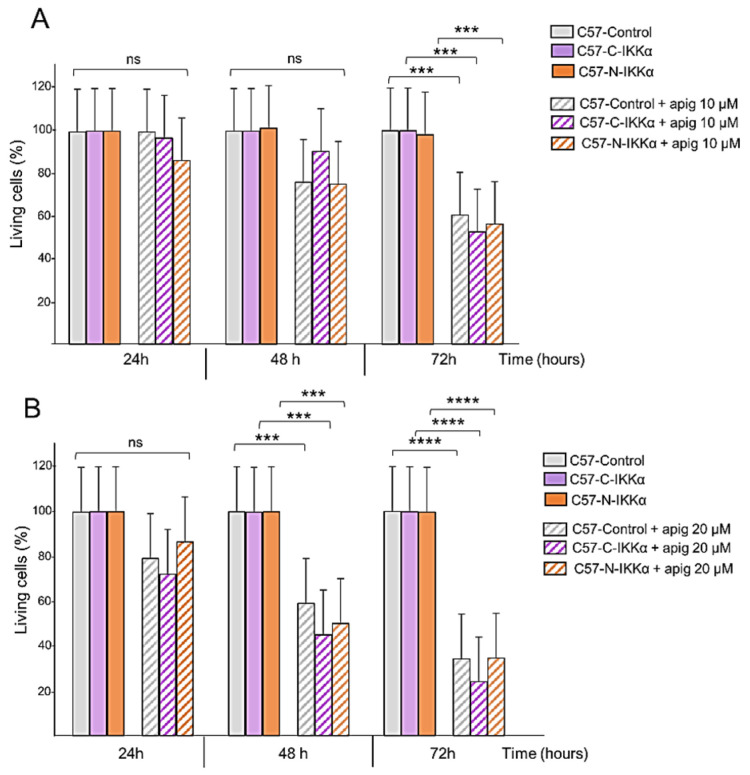
Effect of apigenin on C57 skin carcinoma cells of the three genotypes. (**A**,**B**) Apigenin treatment causes cell growth inhibition in C57 cells of the three genotypes. Percentage of cells of the three genotypes at the indicated times in the absence or presence of 10 µM (**A**) and 20 µM (**B**) of apigenin at the indicated hours of treatment. Observe that treatment with both doses of apigenin causes minimal changes in the viability of the cells after 24 h of treatment; at 48 h, 20 µM of apigenin caused a reduction of cell viability (*p* < 0.0001); at 72 h of treatment a marked reduction in cell viability was detected with both doses of apigenin tested in the C57 cells of the three genotypes (*** *p* < 0.001; **** *p* < 0.0001). Data are shown as mean ± SEM by t-test.

**Figure 9:**
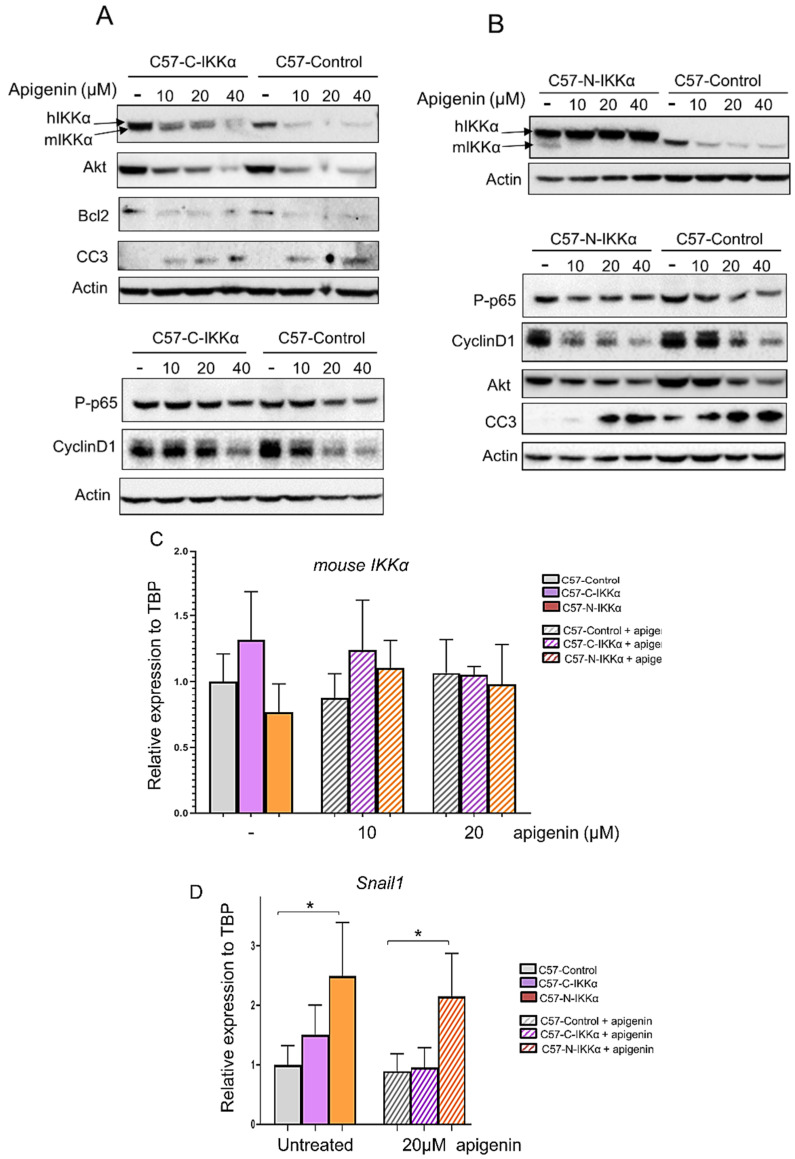
Apigenin inhibits the expression of cytoplasmic but not nuclear IKKα protein. Impact of apigenin on the expression other tumoral marker proteins. (**A**,**B**) WB showing the changes induced by 10–40 µM of apigenin in C57 cells of the three genotypes after 48 h of treatment. (**A**) WB showing that apigenin inhibits the expression of the exogenous IKKα localized in the cytoplasm of C57-C-IKKα cells, as well as the endogenous IKKα. (**B**) WB showing that apigenin diminishes the levels of endogenous IKKα in both C57-Control and C57-N-IKKα cells upon 48 h of treatment with either 10, 20 or 40 µM of apigenin, but does not affect exogenous IKKα localized in the nucleus. Apigenin also attenuates the expression of Cyclin D1, Bcl2 and Akt proteins and increases the cleaved-Caspase-3; a faint attenuation of P-p65 was noticed in the three types of C57 cells (**A**,**B**). Since C57-N-IKKα cells express increased levels of the transgene, and to allow comparison of the expression of IKKα in the same blot, the amount of total protein extracts loaded was 20 µg in the case of C57-N-IKKα cells and 50 µg of C57-Control and C57-C-IKKα cells. (**C**) qPCR analysis of the expression of mouse *IKKα* mRNA in C57-Control and C57-C-and-N-IKKα. Note that apigenin does not inhibit the expression of the endogenous *IKKα* at transcriptional level. Cells were incubated in the absence or presence of 10–40 µM apigenin for 48 h. (**D**) qPCR analysis of the expression of mouse *Snail1* mRNA in C57-Control and C57-C-and-N-IKKα cells in the presence or absence of apigenin 20 µM. Observe that apigenin does not inhibit the increased expression of *Snail1* in the C57-N-IKKα cells. Data are shown as mean ± SEM; * *p* < 0.05 by two-tailed unpaired t test with Welch’s correction.

The capacity of apigenin to inhibit NMSC has been reported [35,36]; however, its mechanisms of action remain largely unknown. In particular, whether apigenin has an effect on IKKα expression has not been studied yet. Therefore, we proceeded to analyze this. First, we performed cell viability assays of cells growing in the absence or presence of apigenin at two different concentrations, 10 and 20 µM, which are the concentrations reported in different types of tumors, including in NMSC [35,36,47], for different periods (24–72 h). Then, 10 µM apigenin resulted in only a slight reduction in the number of cells in the three types of C57 clones at 72 h (Figure 8A). However, when cells were grown in the presence of apigenin 20 µM, a significant reduction in the number of cells was detected from 48 h of culture and even more at 72 h (Figure 8B). Then, we explored whether, as reported in prostate cancer cells [33], apigenin regulates IKKα in skin cancer cells. We analyzed by WB the possible influence of apigenin treatment in the expression levels of IKKα in the C57 cells of the three genotypes. Our results showed that treatment with 10–40 µM of apigenin during 48 h was enough to diminish the expression of endogenous mouse IKKα in the three types of C57 cells (Figure 9A,B); at this time point, apigenin also reduced the levels of the exogenous cytoplasmic IKKα in the C57-C-IKKα cells at the three concentrations tested (10–40 µM) (Figure 9A); however, levels of exogenous nuclear IKKα remained unaltered in the presence of apigenin 10–40 µM (Figure 9B), suggesting that the regulation of IKKα by apigenin is post-transcriptional and cannot be exerted when IKKα is localized in the nuclear compartment. To further discard the possible involvement of a transcriptional process in the apigenin-induced inhibition of IKKα, endogenous mouse IKKα mRNA expression was assessed in the three types of C57 cells. The results showed that apigenin did not diminish mouse IKKα expression at 48 h of culture (Figure 9C). Thus, these results suggested that apigenin regulated IKKα expression levels at post-transcriptional level. Next, we analyzed whether apigenin was able to attenuate the molecular changes induced by the exogenous IKKα, which are related to tumor development and progression of cutaneous SCCs. Our results showed that even the lowest concentration of apigenin tested (10 µM) was able to reduce the expression of Akt, Bcl2 and Cyclin D1 and caused the appearance of cleaved-Caspase 3 (CC3) in the three types of C57 cells; furthermore, it was observed that the changes in the expression levels of these proteins were dose-dependent, being more extreme at 40 µM (Figure 9A,B). Since, in prostate cancer cells, the antitumor effect of apigenin was reported to be mediated by its ability to directly interact with IKKα and suppress NF-κB/p65 activation, we analyzed the activation of the NF-κB/p65 subunit (measured as levels of P-p65) in the three types of C57 cells. We found a slight attenuation of NF-κB/p65 activation for 10–40 µM of apigenin tested in the three types of C57 cells (Figure 9A,B), which suggests that in skin carcinoma cells the anti-tumor effect of apigenin is not principally mediated by the inhibition of the NF-κB/p65 pathway. Additionally, we analyzed whether the treatment of C57 cells with apigenin had any effect on the expression levels of *Snail1* mRNA. As expected, given the lack of effect of apigenin on nuclear IKKα, we did not find a decrease in the expression levels of *Snail1* in C57-N-IKKα cells (Figure 9D), which remained significantly induced in these cells.

## 3. Discussion

We have analyzed the mechanisms through which IKKα favors mouse cutaneous SCCs aggressiveness. Recent results from our group and others [5,15,16,17] have underscored the relevance of studying the nuclear or cytoplasmic localization of IKKα for the understanding of the mechanisms of the protumoral role of IKKα in NMSC and other types of cancer [15]; therefore, here, we have generated variants of the PDVC57 skin carcinoma cells that express exogenous IKKα in the nucleus or in the cytoplasm. We have found that IKKα confers a greater capacity of migration to the cells in both subcellular localizations, being this effect especially remarkable in the case of the C57-N-IKKα cells. Additionally, C57 cells expressing ectopic IKKα showed increased apoptosis resistance and augmented capacity of survival when they are grown under stress, such as in the absence of serum, i.e., while C57-Control cells showed a strong cleaved-Caspase 3 band (an indicator of apoptosis), there was less amount of cleaved-Caspase 3 in the C57-C-and -N-IKKα cells, which in contrast exhibit greater expression of the prosurvival proteins Bcl2 and Akt. Overexpression of both Bcl2 and Akt has been found in different types of tumors and is associated with EMT, increased invasiveness [48,49,50] and resistance against EGFR-TK inhibitors [51]. The acquisition of migratory properties is a hallmark of cells undergoing EMT changes, which can also be accompanied by a switch toward a mesenchymal phenotype, as occurs in the C57-N-IKKα cells; the increase in the apoptosis resistance and the augmented capacity of survival is another feature of the EMT as well [52]. Therefore, our results suggest that regardless of its subcellular localization, IKKα increases the malignancy of mouse epidermal SCC cells through molecular changes that resemble those of cells undergoing EMT. In addition, we have proven that in another cell line of murine tumor keratinocytes (PB), the C-and-N-IKKα cells share similar alterations to those found in the C57 cells expressing the exogenous IKKα. This finding is in agreement with the role of IKKα in oral SCCs and breast cancer, in which it was reported that IKKα mediated the EMT process induced by TGF-β1, contributing in this way to the tumor-promoting role of this growth factor [53,54]. In addition, in the case of non-small cell lung cancer (NSCLC), our group and others found that IKKα increased the migration of NSCLC cells and promoted NSCLC progression [44,55]. Accordingly, the upregulation of IKKα has been associated with decreased patient survival, and it has been suggested that IKKα levels could overall predict poor clinical outcome in lung adenocarcinoma patients [55]. Thus, given the relevant role that IKKα plays in the progression of different types of human cancer, further studies on the role of C-and-N-IKKα in human tumor keratinocyte cell lines or skin tumors are necessary.

Although our data showed an enhancement of the expression of markers of tumor progression as a consequence of the ectopic expression of IKKα in both localizations, this is even more outstanding in C57-N-IKKα cells, which in addition to the increased levels of MMP9, Akt, Bcl2 and the decreased detection of cleaved-Caspase 3, they induce the expression of c-Myc, that in turn may contribute to the EMT process [43]. Additionally, the overexpression of IKKα in the nucleus of C57 cells causes an acquisition of a marked fibroblastoid phenotype and induces the transcription of *Snail1* (which has a pivotal role in the EMT process [56]) and *Vimentin*, and represses the expression of K5; therefore, the switch in the expression of the epithelial marker K5 toward that of Vimentin correlates with the morphological change detected in the shape of C57-N-IKKα cells [56]; the induction of *Snail1* by nuclear IKKα was also observed in NSCLC cells [44]. Altogether, these changes make C57-N-IKKα cells appear as potentially more aggressive, since *Snail1* is considered a determinant of the progression of carcinomas [27], c-Myc expression is found in skin SCCs of poor prognosis [57], and the downregulation of the expression of keratins, including that of K5 in skin tumors, is associated with increased malignancy and conversion to spindle cell carcinomas [58].

Therefore, our results here showing that the expression of nuclear IKKα in C57 cells worsen the malignant characteristics of tumor keratinocytes are in agreement with our previous findings in chemical skin carcinogenesis experiments in transgenic mice, which showed that non-tumorigenic keratinocytes expressing increased levels of IKKα in the nucleus lead to an enhanced progression of SCCs [15]; they also agree with results showing that in human patients, skin SCCs expressing higher levels of nuclear IKKα are more aggressive and present higher risk of metastasis [5]. Furthermore, this correlation between nuclear IKKα expression and the worsening of tumor characteristics also applies to lung adenocarcinomas, in which a correlation was found between increased levels of nuclear IKKα and a poor prognosis and lower survival of patients [55,59].

As mentioned, skin SCC is a common type of neoplasia, representing an important problem on patients’ life and clinical management. Although they do not metastasize frequently, once they do, SCCs display considerable aggressiveness, leading to the death of affected individuals and making it necessary to find an effective treatment for this type of cancer. For this reason, we have carried out experiments aimed at further analyzing the mechanisms of action of apigenin, a flavonoid known for its anti-cancer function in diverse types of tumors. In the case of cutaneous SCCs, recently, it has been reported that apigenin inhibits UVB and DMBA/TPA skin carcinogenesis [35,36,60,61]; however, the mechanisms through which it exerts this effect remain largely unknown. In particular, the possible involvement of IKKα in the anti-tumor role of apigenin in NMSC inhibition has not been addressed yet. This is an important issue since, in prostate cancer cells, it was shown that apigenin acted as an anti-cancer agent through its binding to IKKα, which triggered the antitumor response [33].

Here, we have found that apigenin diminishes the expression levels of the ectopic IKKα protein in the cytoplasm but not in the nucleus of C57 cells. This is an interesting result since it was described that the regulation of IKKα by apigenin occurred both in the cytoplasm and in the nucleus of PC3-tumor prostate cells [33]; moreover, in contrast to the results obtained in prostate cancer cells in which one of the main mechanisms of action of apigenin is the IKKα-mediated inhibition of the activation of NF-κB/p65, we have found that, in the C57 cells, apigenin does not inhibit the activation of this signaling pathway in a relevant way, even at high concentrations of apigenin (20–40 µM). On the contrary, we have found that apigenin attenuates the expression of proteins recognized as key molecules for skin cancer progression, such as Cyclin D1, Akt and Bcl2, while inducing the cleaving of Caspase 3, which plays a central role in apoptosis. Additionally, our results also show that apigenin downregulates IKKα at post-transcriptional level, since we did not find differences in the expression of the endogenous mouse *IKKα* mRNA (the expression of the ectopic IKKα is under the control of the β-actin promoter; thus, it is not subjected to the regulatory elements of the endogenous *IKKα* gene). Our results also complement those reported by other groups which described that apigenin exerted, at least in part, its anticancer effect in the nucleus of cells. It was reported that apigenin interacted with DNA in prostate cancer cells [62] and that it was involved in mRNA metabolism/splicing in the nucleus of breast cancer cells [63]. Therefore, further research work is needed to investigate whether the mechanism of action of apigenin is cell-type specific, since in our models of PDVC57 cells apigenin does not affect nuclear IKKα, while it is able to diminish levels of cytoplasmic IKKα. It would be interesting to study the mechanism/s through which this reduction takes place, i.e., whether there is a direct interaction apigenin-IKKα or if the formation of a complex with another molecule is required.

Therefore, our findings are noteworthy as they suggest that apigenin could be a promising anti-NMSC drug therapy since, in addition to reducing the levels of IKKα, which favors skin SCC progression, it reduces those of Cyclin D1, Akt and Bcl2, which also play a relevant role in NMSC malignization. However, it has been described that cutaneous SCC with higher risk to metastasize express higher levels of nuclear IKKα [5]; thus, it would be interesting to study whether these aggressive tumors, in addition to elevated levels of nuclear IKKα, also express high levels of cytoplasmic IKKα, and whether the changes in the expression levels of the aforementioned proteins -Cyclin D1, Bcl2, Akt- by apigenin are dependent or not in the reduction of the cytoplasmic IKKα levels.

In summary, our model of C57 skin carcinoma cells that express ectopic IKKα in the nucleus is a valuable model to study the molecular mechanisms that lead to the enhanced aggressiveness of cutaneous SCCs with high risk to metastasize (that contains elevated levels of nuclear IKKα). In addition, our data suggest that IKKα may be a therapeutic target for the treatment of skin SCCs and that apigenin could be a potential anti-NMSC cancer treatment. Moreover, and given the relevance of the subcellular localization of IKKα for the development of NMSC, our model of C57-C-and-N-IKKα cells provides a powerful tool to analyze whether IKKα inhibitors are capable of targeting IKKα when localized to a specific subcellular compartment.

## 4. Materials and Methods

### 4.1. DNA Constructs

The C-and-N-IKKα constructs have been previously described [15]. Briefly, both of them contain the sequence of the human *IKKα* gene cDNA, but the N-*IKKα* construct has an extra NLS (nuclear localization signal) in 5′; in contrast, in the C-IKKα construct, the internal NLS site was removed. Both constructs were subcloned in the pRC vector containing the β-Actin promoter (25) and the Flag-tag. The empty pRC-β*Actin* vector was used as a Control (C57-Control cells). All constructs confer resistance to G418.

### 4.2. Cells, Culture Conditions and Transfection Assays

PDVC57 and PB cells were provided by Dr. Miguel Quintanilla (Instituto de Investigaciones Biomédicas (IIBM), Madrid, Spain. They were cultured in DMEM-10% FBS. They were permanently transfected using the calcium phosphate method and selected using G418 (0.45 mg/mL, BioNova, Fremont, CA, USA).

### 4.3. Colony Forming Assays

A total of 5 × 10^2^ and 1 × 10^3^ cells were seeded per duplicate in DMEM-10% FBS in p100 plates. Medium was replaced every 2 days. Growing colonies were photographed at different time points. Experiments were performed three times per duplicate.

### 4.4. Wound Healing Assays

Cells growing in monolayer cultures were incubated 2 h at 37 °C in DMEM plus 5 mg/mL of mitomycin C; then washed with PBS. After 1 h, a scratch wound was created in vitro by scraping the cell monolayer with a sterile pipette tip. Healing was measured at 48 h post-scratch. Three experiments were performed by duplicate.

### 4.5. Growth in Serum-Free Medium

Next, 2 × 10^6^ cells were seeded into p100-plates in complete medium. After 24 h, the medium was replaced by serum-free DMEM. Cells were collected 7 days later. Three experiments were performed by duplicate.

### 4.6. Cell Viability Assay

The effect of apigenin on the viability of C57 cells of the three genotypes was evaluated. Next, 5 × 10^4^ cells were seeded onto p60 plates in complete medium (DMEM-10% FBS). Following incubation overnight, the cells were treated with 10 or 20 µM apigenin in dimethyl sulfoxide (DMSO) for 24, 48 and 72 h in a humidified atmosphere of 5% CO_2_ at 37 °C. Following incubation for the indicated times, cells were trypsinized and counted with a Neubauer’s chamber. Three experiments per duplicate were performed. The results were expressed as a percentage of growth.

### 4.7. Immunofluorescence Staining

Cells grown in coverslips were fixed in methanol/acetone (1/1). Primary antibodies used were: anti-Flag antibody (F3040, Sigma-Aldrich, St. Louis, MO, USA); anti-K5 (PRB-160P) (Biolegend, San Diego, CA, USA); and anti-IKKα (NB100-56704, Novus Biologicals, Cambridge, UK).

### 4.8. Western Blot Analysis

Total protein extracts (40 μg) were subjected to SDS/PAGE. The separated proteins were transferred to nitrocellulose membranes (Amersham, Arlington Heights, IL, USA; BioRad, Marnes-la-Coquette, France) and probed with antibodies against IKKα (NB100-56704) (Novus Biologicals, Cambridge, UK); c-Myc (626802; Biolegend, San Diego, CA, USA); Actin (6276; Abcam, Cambridge, UK); GAPDH (sc-32233; Santa Cruz Biotechnology Inc. Europe, Heidelberg, Germany); MMP9 (AB19016; Merck Millipore, Darmstadt, Germany); Bcl2 (3498), cleaved-Caspase 3 (9661), Akt (4691) and P-p65 (3039) (Cell Signaling Technology, Danvers, MA, USA); Cyclin D1 (MA5-16356; Invitrogen, Thermo Fisher Scientific, Waltham, MA, USA). In all cases, samples were subjected to luminography with the Supersignal West Pico Chemiluminescent Substrate (Pierce Biotechnology Inc., Rockford, IL, USA) or with Clarity Western ECL Substrate, 1705061 (BioRad, Marnes-la-Coquette, France).

### 4.9. RNA Isolation and Quantitative RT-qPCR Analysis

Total RNA from C57 cells of the three genotypes was isolated using miRNeasy Mini Kit (Qiagen), and DNA was eliminated with an Rnase-Free DNase Set (Qiagen, Hilden, Germany) according to the manufacturer’s instructions. The reverse transcription reaction was performed using the High Capacity cDNA Reverse Transcription Kit (Applied Biosystems, Waltham, MA, USA) for mRNA. Quantitative qRT-PCR was performed in a 7500 Fast Real Time PCR System using GoTaq qPCR Master Mix (Promega, Maddison, WI, USA) for mRNA. The sequences of the oligonucleotides specific for Snail1 and mouse IKKα used are listed in Table 1. TBP was used as a housekeeping for normalization of gene expression. Fold changes were calculated using the efficiency-corrected model [64], given by the formula Ratio = (E_TARGET_)^∆Ct^_TARGET_/(E_REF_)^∆Ct^_REF_, being E the amplification efficiency of the target or the reference genes, Ct the cycle threshold and ΔCt_TARGET_ is the Ct difference of a treated and control sample for the target gene (i.e., ΔCt_TARGET_ = Ct_CONTROL_−Ct_TREATED_), and ΔCt_REF_ is the Ct difference of a treated and control sample for the reference gene (i.e., ΔCt_REF_ = Ct_CONTROL_−Ct_TREATED_).

## Figures and Tables

**Table 1:** Oligonucleotides sequences used for the quantitative analysis of the expression of *Snail1*, mouse *IKKα* and *Vimentin* genes.

Oligonucleotides	Sequence
Snail1 (forward)	CTTGTGTCTGCACGACCTGT
Snail1 (reverse)	GGAGCAGGAGAATGGCTTC
mIKKα (forward)	CCCTCCAGTATCAGCATGGC
mIKKα (reverse)	GTGCTAACGTCTCTCACACA
Vimentin (forward)	CCAACCTTTTCTTCCCTGAAC
Vimentin (reverse)	TTGAGTGGGTGTCAACCAGA
TBP (forward)	GGGAGAATCATGGACCAGAA
TBP (reverse)	GATGGGAATTCCAGGAGTCA

## Supplementary Materials

The following supporting information can be downloaded at: www.mdpi.com/article/10.3390/ijms23031375/s1.

## Abbreviations

BCCs	basal cell carcinomas
CC3	cleaved-Caspase 3
EMT	epithelial–mesenchymal transition
FBS	fetal bovine serum
NMSC	non melanoma skin cancer
NSCLC	non-small cell lung cancer
RDEB	recessive epidermolysis bullosa
SCCs	squamous cell carcinomas
