# Supplementary figures and images for: IKKα Induces Epithelial–Mesenchymal Changes in Mouse Skin Carcinoma Cells That Can Be Partially Reversed by Apigenin
Source: Int J Mol Sci. 2022 Jan 25;23(3):1375. doi: 10.3390/ijms23031375 (PMC8836221; doi:10.3390/ijms23031375)

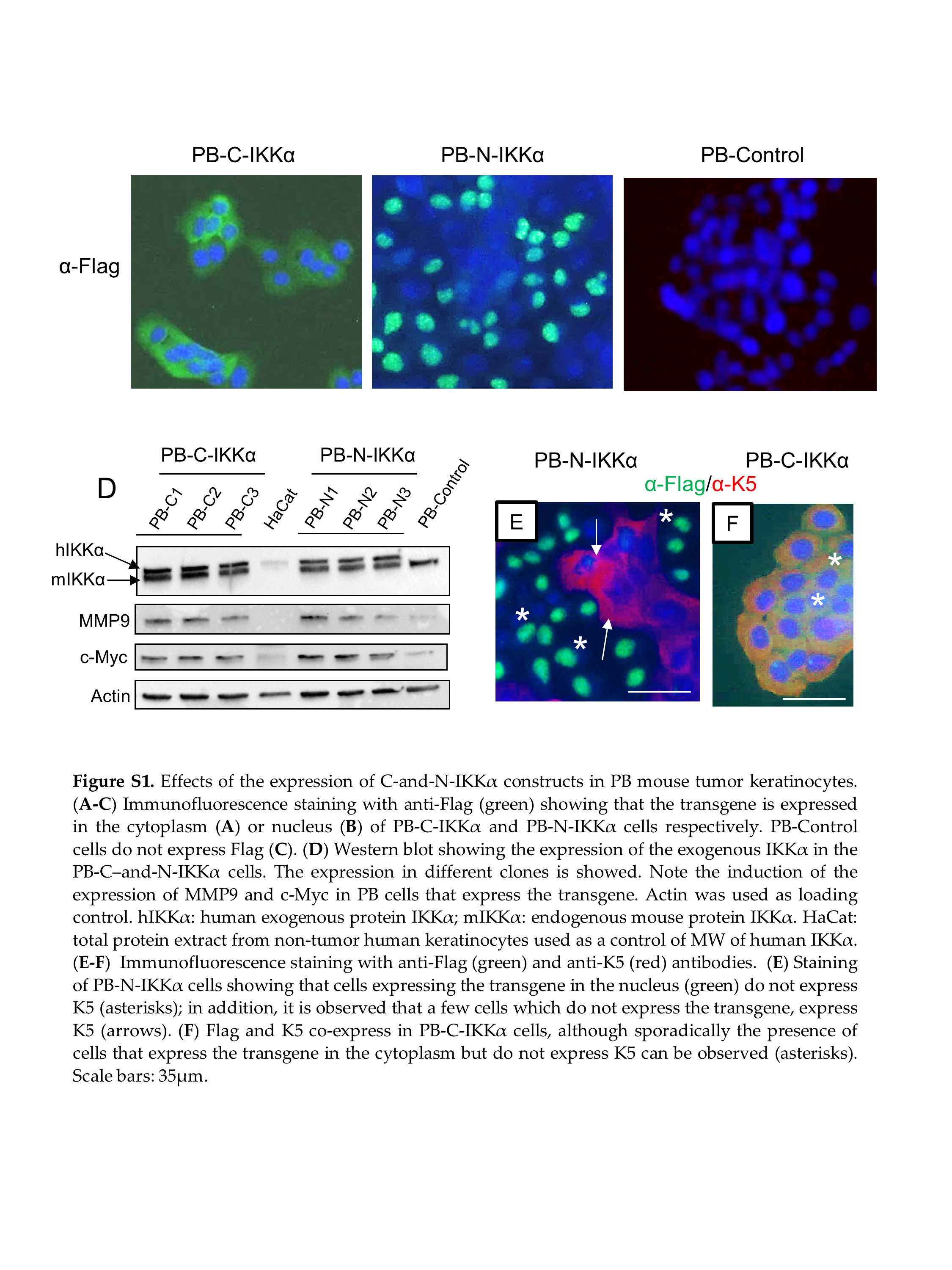

Supplement: Supplementary file 1 [file ijms-23-01375-s001.zip › ijms-1540687-supplementary.tif]
